# Training the MCH workforce: the Time for Change is now

**DOI:** 10.1007/s10995-022-03438-x

**Published:** 2022-08-18

**Authors:** Jonathon P. Leider, Jamie Stang, Zobeida E. Bonilla, Jason Orr, Christine M. Plepys, Moriah Gendelman, Ellen W. Demerath

**Affiliations:** 1 School of Public Health, University of Minnesota, Mayo A301, 420 Delaware St SE, 55455 Minneapolis, MN United States; 2 Association of Schools and Programs of Public Health, Washington, DC United States; 3 de Beaumont Foundation, Bethesda, MD United States

**Keywords:** Public health workforce, MCH workforce, Academic public health, Graduate outcomes

## Abstract

**Introduction:**

Maternal and child health (MCH) services are critical for vulnerable populations. Workforce shortages, poor retention, and gaps in necessary trainings impede the capacity of public health systems to address needs. This manuscript characterizes the current MCH workforce, MCH program applicants and graduates, and describe findings within a national context to devise elements of a recruitment and retention strategy.

**Methods:**

Data were obtained for public health program applicants, first-destination employment outcomes, and worker perceptions and demographics. Data were stratified according to the MCH and total public health workforce and by local, state, and national totals. Data were characterized by degree type, discipline, demographics, and employment outcomes.

**Results:**

MCH staff constitute 11% of the state and local governmental public health workforce. MCH staff are approximately as diverse, have higher educational attainment, and are more likely to hold nursing degrees than the rest of the public health workforce. Yet, just 14% of MCH staff hold any type of public health degree. The MCH pipeline from academia appears modestly sized, with approximately 5% of applicants between 2017 and 2021 applying to a MCH master’s degree.

**Discussion:**

The MCH workforce has a lower proportion of formal training or degrees in public health, though trends seem to indicate improvements. However, it is critical that a multi-faceted recruitment and retention strategy be coordinated by a broad range of stakeholders. These efforts will serve to improve the capability and capacity of the public health system to address critical needs of increasingly diverse MCH populations.

**Significance:**

In order to modernize and reimagine the academic-public health pipeline, it is critical to better understand how many applicants and graduates exist within Maternal and Child Health programs across the US, and their characteristics. This manuscript connects that information with the most recently available public health workforce information on demographics, workplace perceptions, and intent to leave among staff at state and local health departments. Data presented in this paper allow the most comprehensive characterization of the MCH academia->practice pipeline to-date, identifies a fundamental disconnect in those career pathways, and offers options to repair that break.

## Introduction

Maternal and child health (MCH) services address the needs of the most vulnerable populations: pregnant women, infants, children, adolescents, and their families. These needs are met through the coordinated efforts of the Health Resources and Services Administration (HRSA) Maternal and Child Health Bureau (MCHB), the federal agency that administers funding for programs, research, and workforce development to address the needs of the MCH population. This is accomplished in partnership with departments of public health, local communities, and non-profit organizations at the federal, state, and local levels (MCHB, 2021). While these services aim to meet the unique needs of MCH populations, shortages in the MCH workforce and lack of training opportunities create gaps in the provision of needed services (Perlino, 2006; Yeager et al., 2016). Deficits in the public health workforce were foreshadowed by the Associations of Schools and Programs in Public Health (ASPPH) over ten years ago (Association of Schools of Public Health [ASPH], 2008).

As illustrated by the life course health development model (Halfon et al. 2014), MCH populations are considered especially vulnerable and in need of support due to the sensitivity of pregnancy, infancy, and childhood to biological, environmental, and social exposures, which have particular potency to shape health trajectories over the subsequent life course. The ability to develop, administer and evaluate effective programs for MCH populations requires training that integrates the biological and social constructs of health and development, attention to multigenerational health, and skills in translating these sciences into family-centered, culturally appropriate services.

Training in public health with a deliberate and intensive focus on the unique needs of MCH populations enables professionals to be better positioned and equipped to work with MCH populations and deliver Title V services. Yet, there is a disconnect between the governmental public health workforce and academia (Yeager et al., 2016); a disconnect that needs to be addressed. This discordance is also present specifically in the governmental MCH workforce and academia. Even before the challenges brought to the fore by the global coronavirus (COVID-19) response, MCH practitioners—a substantial component of the overall public health workforce—have faced serious barriers to achieving adequate capacity (Sellers et al., 2019). Only a small fraction of the state and local MCH workforce (14%) have received a formal public health degree, as observed in findings from the 2017 Public Health Workforce Interests and Needs Survey (PH WINS; de Beaumont Foundation, 2019). The longevity of the MCH workforce is also a point of concern, with substantial gaps anticipated in the future MCH workforce. Analysis of the 2017 PH WINS survey by the Association of Maternal & Child Health Programs (AMCHP) found that many within the MCH workforce report planning to leave their agency (28%) or plan to retire (24%), respectively (AMCHP, 2019; de Beaumont Foundation, 2019). At the same time, the U.S. now graduates over 35,000 bachelors, masters, and doctoral students each year in public health broadly, though only a small fraction (17%) go into governmental public health following graduation (Plepys et al., 2021). These ‘first-destination’ outcomes for recent graduates have been characterized for public health graduates, though relatively little has been published about MCH graduates, including how many graduates there are each year.

To weather the shifting tides of maternal and child health, AMCHP developed a strategic plan (2020) that has identified several priorities for MCH practitioners to address in order to improve the mental and physical health status of MCH populations. While infant mortality in the U.S. has declined over the past decade (NCHS, 2021), maternal mortality has increased (Reproductive Health, 2021) and the United States continues to experience worse outcomes in both MCH indicators compared to other Organization for Economic Co-operation and Development (OECD) countries (World Bank Group, 2020). In particular, African American and American Indian communities experience higher rates of preterm birth and low birth weight, two risk factors for infant mortality. The lack of MCH services in rural areas is another structural barrier to optimal maternal-fetal outcomes. Increasingly, research shows that structural factors and racism play a central role in creating and maintaining disparities in MCH health outcomes. Thus, AMCHP has prioritized addressing racism as a top public health need (AMCHP, 2020). Other MCH-specific priorities include the need for a workforce that can prepare for and respond to public health emergencies, such as the COVID-19 pandemic, assuring availability of health care coverage to all individuals and families during economic downturns, and creating systems of care that meet the needs of a growing number of children and youth with chronic mental and physical health conditions. Thus, strengthening MCH programs that focus on community-based, family-centered care for the most vulnerable populations, such as the Maternal, Infant, and Early Childhood Home Visiting (MIECHV) Program, pregnancy and postpartum services through Medicaid, and the Children’s Health Insurance Program, are additional priorities that require a well-trained MCH workforce (Health Resources & Services Administration 2019, Margolis et al. 2013). Part of the challenge in meeting priorities for the MCH workforce may be compounded by the lack of representativeness with respect to racial and ethnic diversity in the health professions (Salsberg et al., 2021; Grumbach & Mendoza, 2008; Jackson & Gracia, 2014). While the workforce generally reflects the U.S. population, this may not be the case in many agencies or geographies. As described by Grumbach & Mendoza (2008), the workforce does not reflect the populations served across all agency types within MCH. MCH is not alone in this challenge; a recent survey of gender, race, and ethnic diversity of U.S. health occupations found that Blacks, Hispanics, Native Americans, and Pacific Islanders were underrepresented in all health professions (Salsberg & Westergaard, 2021; National Center for Health Workforce Analysis [NCHWA], 2017).

In this manuscript, we illustrate the demographic and educational characteristics of the MCH workforce with respect to the nation (all local and state MCH staff) and the aggregate national workforce, identifying some disconnects between the supply and demand for MCH workers. Educational diversity of the workforce is discussed from the standpoints of degrees attained and disciplines that those degrees represented, with specific attention paid toward formal public health education. Finally, recommendations are offered to top executives in governmental public health that may serve to enhance recruitment and retention of an adequate MCH workforce.

## Methods

Data used in analyses in this manuscript came from three primary sources: 2017 PH WINS data, ASPPH first-destination employment outcomes data, and ASPPH applicant data from their centralized application platform (SOPHAS) in which programs self-identify their program categories and an identifier is present within the dataset for MCH. PH WINS is nationally representative, using a complex sampling design and employs balanced repeated replication weights to account for this design and non-response (Leider et al., 2019). PH WINS data were collected in 2017 from approximately 47,000 respondents, including 5,500 staff working in MCH, including self-reported MCH workers, WIC staff, and Family Planning staff. As part of characterizing educational attainment of the state and local MCH workforce, authors coded the subject or major of respondents’ degrees according to predefined subjects and professional degrees, where identifiable. Descriptive statistics were used to characterize the current MCH state and local public health workforce according to job classification, highest degree obtained, race and ethnicity, tenure in the public health workforce, and respondents’ intent to leave or retire. Educational attainment was then characterized. Each analysis compared MCH staff against the total state and local governmental public health workforce.

Program applicant data were obtained from SOPHAS for Master of Public Health (MPH) degree applicants across the 2017–2021 academic years. Applicants to degree programs associated with ASPPH’s designated MCH subject areas and emphases were compared against all national public health program applicants. SOPHAS includes applicants from 115 schools and programs, comprising 76% of all public health graduates nationally. Descriptive statistics were provided around the MCH and national applicant totals according to gender, citizenship, race and ethnicity, and age at application. Graduation outcomes data were obtained from ASPPH for public health graduates across the 2017–2019 academic years, with outcomes data from up to one-year post-graduation (“first-destination outcomes”). Data collection methods are described in detail elsewhere (Plepys, 2021). Employment sector by degree level was reported. Data were analyzed in Stata 16.1 (StataCorp LLC, College Station, Texas).

## Results

### Characterizing the current MCH state and local health department workforce

PH WINS data show that staff working in the maternal and child health program area within state and local health departments look distinct from each other and from the overall governmental public health workforce (Table 1 and Appendix Table 1). Among MCH staff that work in state health agency central offices (SHA), 40% work in job classifications falling under the public health sciences, compared to 16% of MCH staff in local health departments (LHD) and 33% of all public health staff (including MCH) nationally. Moreover, about 53% of the MCH workforce locally works in occupations classified as clinicians or laboratorians, compared to 22% among MCH SHA staff and 25% of all staff nationally.

**Table 1:** Composition of governmental public health workforce (state and local MCH practitioners vs. national)

	MCH (%)			National (%)
	**State (weighted n = 4,347)**	**Local** **(weighted n = 16,066)**	**Total** **(weighted n = 20,413)**	**Total** **(weighted n = 179,587)**
***Job classification***				
Administration	33	26	**27**	**40**
Clinical/Laboratory	22	53	**47**	**25**
Public Health Sciences	40	16	**21**	**33**
Social Services / All Other	4	5	**5**	**2**
***Highest Degree attained***				
No college	7	16	**14**	**18**
Associates	6	16	**13**	**15**
Bachelor’s	32	42	**40**	**36**
Master’s	48	24	**29**	**26**
Doctoral	6	2	**3**	**5**
***Race/Ethnicity***				
American Indian / Alaskan Native	1	0	**0**	**0**
Asian	3	4	**4**	**5**
Black / African American	17	14	**15**	**16**
Hispanic / Latino	9	17	**16**	**13**
Native Hawaiian or Pacific Islander	0	0	**0**	**0**
White	64	57	**59**	**59**
Two or more races	6	6	**6**	**6**
***Tenure in Public Health Practice***				
0–5 years	24	31	**30**	**30**
6–10 years	22	19	**19**	**18**
11–15 years	14	15	**15**	**16**
16–20 years	14	12	**13**	**14**
21 + years	26	23	**23**	**21**
***Intent to Leave or Retire***	46	41	**42**	**41**

Data source: PH WINS 2017. Estimates are shown as percents

Overall, the MCH workforce is approximately as racially and ethnically diverse as the workforce overall, with 36% of state health agency staff identifying as Black, Indigenous, and People of Color (BIPOC), compared to 43% locally and 41% among all staff. Notably, significant differences in diversity are observed between MCH staff working locally within so-called “big cities” (71% are BIPOC) compared to other LHDs (36% are BIPOC). Racial and ethnic diversity among management/executive positions is similarly distributed. Among State MCH managers and executives, 37% are BIPOC, as are 42% of local managers and executives, compared to 37% of all managers/executives nationwide. Among big city health departments only, 62% of local managers and executives are BIPOC.

The MCH workforce is more highly educated overall compared to all staff nationally, with 87% of MCH SHA-CO employees with a bachelor’s or higher, 68% of MCH local staff, and 68% of all staff nationally. Relatively more MCH staff have formal education in nursing, including 14% of the MCH SHA workforce, 30% of MCH LHD, and 19% of all staff nationally (Table 2 and Appendix Table 2). Approximately 26% of the MCH SHA workforce has any type of public health degree, compared to 9% of MCH LHD staff and 14% of all staff. Data from PH WINS suggest MCH staff come to their job from an extremely diverse academic background (Appendix Table 3). Among MCH staff, 43% of associates, 23% of bachelors, 13% of masters, and 10% of those with a doctorate (including MD/JD) reported they studied nursing. 21% of bachelors, 12% of masters, and 2% of doctorates studied nutrition or dietetics. 2% of bachelors, 26% of masters, and 14% of those with a doctorate studied public health.

**Table 2:** Educational attainment of the governmental public health workforce (state and local MCH practitioners vs. national)

	MCH (%)			National (%)
	**State** **(unweighted n = 1,495)**	**Local (unweighted n = 3,903)**	**Total** **(unweighted n = 5,428)**	**Total (unweighted n = 47,272)**
Assoc Nursing	5	12	**10**	**9**
Assoc Other	11	15	**14**	**18**
BSN	10	20	**18**	**11**
BSPH	0	0	**0**	**0**
Bachelors other	62	43	**47**	**47**
MPH	19	5	**8**	**8**
MSN	3	4	**4**	**2**
MSW	3	2	**3**	**2**
Master’s other	27	13	**16**	**15**
DrPH/PhD	2	0	**1**	**1**
DNP	0	0	**0**	**0**
Doctoral other (including JD, MD)	5	1	**2**	**4**
***Nursing (any level)***	14	30	**27**	**19**
***Public Health (any level)***	26	9	**13**	**14**

Data source: PH WINS 2017. Estimates are shown as percents

**Table 3:** Master’s applicant profiles of the governmental public health workforce (MCH practitioners vs. national)

	MCH (%)					National (%)				
**Gender**	**2017 (n = 1,066)**	**2018 (n = 1,033)**	**2019 (n = 757)**	**2020 (n = 860)**	**2021 (n = 1,094)**	**2017 (n = 16,576)**	**2018 (n = 17,588)**	**2019 (n = 16,882)**	**2020 (n = 20,679)**	**2021 (n = 22,127)**
Women	89	88	90	91	93	72	72	71	72	75
Men	10	11	7	7	7	27	27	25	25	25
Other	0	0	3	1	0	0	0	4	2	0
	**MCH (%)**					**National (%)**				
**Citizenship**	**2017 (n = 1,066)**	**2018 (n = 1,036)**	**2019 (n = 768)**	**2020 (n = 916)**	**2021 (n = 1,157)**	**2017 (n = 16,575)**	**2018 (n = 17,579)**	**2019 (n = 16,894)**	**2020 (n = 20,623)**	**2021 (n = 22,048)**
Foreign Citizen	18	18	19	16	14	21	22	26	23	22
US Citizen	82	82	81	84	86	79	78	74	77	78
	**MCH (%)**					**National (%)**				
**Race/ethnicity**	**2017 (n = 817)**	**2018 (n = 823)**	**2019 (n = 605)**	**2020 (n = 770)**	**2021 (n = 988)**	**2017 (n = 12,156)**	**2018 (n = 13,109)**	**2019 (n = 12,150)**	**2020 (n = 15,457)**	**2021 (n = 16,749)**
Asian	12	15	11	11	9	16	17	16	16	17
Black	17	17	25	24	23	16	16	16	18	17
Hispanic / Latino	12	14	12	11	13	11	13	13	13	14
Native American or Alaskan Native	0	0	0	6	6	0	0	1	0	0
Native Hawaiian or Native Pacific Islander	0	0	1	2	1	0	0	0	0	0
White	55	49	46	42	45	52	51	50	49	48
Two or More	4	4	3	4	3	4	4	4	4	4
	**MCH (%)**					**National (%)**				
**Age at application**	**2017 (n = 1,066)**	**2018 (n = 1,029)**	**2019 (n = 738)**	**2020 (n = 846)**	**2021 (n = 1,093)**	**2017 (n = 16,571)**	**2018 (n = 17,504)**	**2019 (n = 16,297)**	**2020 (n = 20,119)**	**2021 (n = 22,078)**
< 22	17	17	23	18	23	17	17	18	17	20
22–26	62	59	54	57	52	52	52	51	50	49
27–34	17	19	18	18	18	21	21	20	21	20
35+	4	5	6	7	7	10	10	10	12	11
	**MCH (%)**					**National (%)**				
**Undergraduate major**	**2017 (n = 1,040)**	**2018 (n = 981)**	**2019 (n = 717)**	**2020 (n = 853)**	**2021 (n = 1,313)**	**2017 (n = 14,983)**	**2018 (n = 16,367)**	**2019 (n = 15,755)**	**2020 (n = 19,433)**	**2021 (n = 21,251)**
Anthropology	4	4	2	3	2	2	2	2	2	2
Health Care Administration	0	0	1	0	1	0	0	2	0	2
Health Sciences	6	8	9	8	5	7	7	7	7	7
Mathematics & Statistics	0	0	0	1	0	0	0	2	2	0
Nursing	2	2	2	4	22	0	2	2	2	2
Nutrition / Dietetics	3	3	2	2	1	2	2	2	2	2
Other	47	45	41	41	34	53	50	47	47	48
Other Biological Sciences	15	15	16	15	16	20	20	19	19	18
Psychology	10	10	7	9	6	7	7	6	6	6
Public Health	10	11	16	16	11	8	9	10	11	12
Sociology	4	3	2	2	2	2	2	2	2	2

Data source: SOPHAS, 2017–2021

Estimates for race/ethnicity are shown among U.S. citizens with reported race/ethnicity

MCH SHA staff have somewhat more experience than their peers, with 76% working in public health practice over five years, compared to 70% of other staff types. 26% of the MCH SHA workforce has worked in public health practice for more than 20 years, compared to 23% of MCH local staff and 21% of all staff nationally. This is particularly relevant, as 46% of MCH SHA staff reported they are considering leaving or planning to retire, compared to 41% of MCH local staff and 41% of all staff nationally.

### Characterizing MCH applicants to and graduates from schools and programs of public health

Between the 2017 and 2021 academic years, 4,694 individuals applied to master’s-level MCH programs using SOPHAS from a cohort of 90 institutions with applicants each year. Among applicants to all master’s programs available in the cohort from SOPHAS (n = 89,291 applicants in 2017–2021), about 5% applied to MCH programs. Approximately 90% of applicants to MCH programs were women, compared to 72% of all applicants. Excepting 2020 and 2021, which saw a decrease in applicants from foreign citizens, approximately 80% of applicants are U.S. citizens, comparable to all applicants to master’s programs in SOPHAS nationally. In 2017, 45% of MCH applicants who were U.S. citizens with reported race/ethnicity identified as BIPOC, compared to 48% of all applicants. In 2021, 55% of MCH applicants were BIPOC, compared to 52% of all applicants. Applicants have a wide variety of undergraduate degrees (Table 3), with other biological sciences, public health, and psychology being the most common among MCH applicants (16%, 13%, and 9%), compared to all undergraduate degree applicants (19%, 10%, and 6%).

**Fig. 1:**
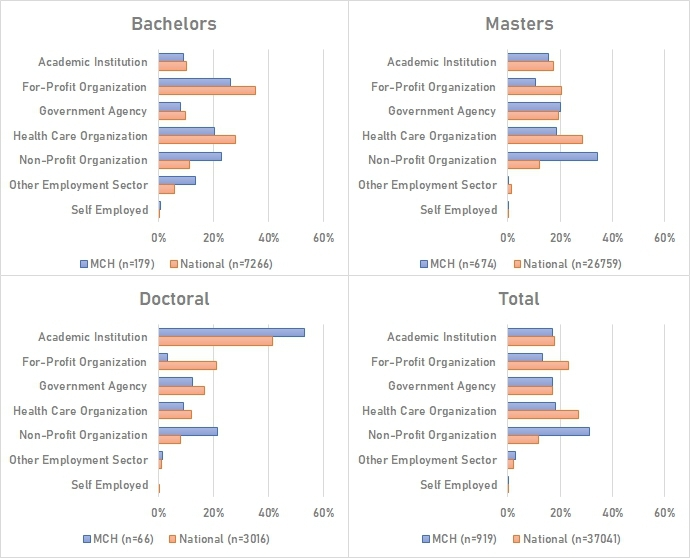
First-destination public health graduate outcomes (MCH practitioners vs. national). Source: Association of Schools and Programs of Public Health. Notes: Charts represent the proportion of known graduate outcomes among n = 37,041 public health graduates for graduating years 2016–2020 that are employed full-time. Employment data are collected up to one-year post-graduation (i.e., reporting years 2017–2021)

Among ASPPH graduates, first-destination employment outcomes were reported for 72,038 students graduating between 2016 and 2019. 7% of MCH graduates were not employed and seeking employment, compared to 5% of all graduates with reported employment outcomes. Among those employed full time (Fig. 1), MCH staff were more likely to work in non-profit organizations (31%) compared to all graduates (12%), and 17% reported working in government at any level, the same as for all reported graduates.

## Discussion

We do not know how many MCH students graduate each year in the United States. Over the past three decades, 400,000 public health degrees have been conferred in the U.S.—137,000 bachelors, 241,000 masters, and 22,000 doctoral degrees (NCES, 2020). Officially, just 2,000 of all public health degrees were from MCH programs (CIP code 51.2209), though this is likely a substantial undercount, as ASPPH reported 246 master’s MCH graduates in 2019, compared to just 92 in the NCES data (NCES, 2020; ASPPH, 2020). One explanation may be a misreporting of MCH graduates as a different type of public health graduate (e.g., “Public Health - General”, 51.2201), though the scale and scope of the underestimate is not well known. While the NCES data capture the ‘universe’ of all public health graduates, an important consideration is that a relatively small proportion of the MCH workforce, as elucidated through PH WINS, has a formal public health degree. This means public health practitioners may not have formal public health education. A data limitation also exists in which the ASPPH and SOPHAS data only represent academic maternal and child health trainings in schools and programs of public health and may not align with the disciplines included as MCH within PH WINS.

What is clear is that the current local governmental MCH workforce has a relatively low proportion of formal training in any public health degree, and that advanced degrees of any type are not equally distributed across the MCH programs in health departments (see Table 1). State MCH staff possess a higher proportion of advanced degrees while local staff possess a higher proportion of nursing associates and bachelor’s degrees with fewer staff trained in MCH and public health. The distribution of educational attainment tracks public health systems theory, wherein local health departments are more likely to deliver direct services (clinical or otherwise), compared to state agencies that deliver more population-based services (Leider et al., 2014). However, that just a minority of state and local MCH staff have any kind of public health degree should be somewhat surprising, given the substantial number of public health degrees now awarded each year. Though the racial and ethnic diversity of the MCH workforce may reflect the MCH populations they serve, equity in public health- and MCH-specific trainings are not well documented. Public health- and MCH-specific trainings are most often, though not exclusively, available at the graduate level, through in-person instruction, and offered during traditional daytime hours. The cost and lack of flexible graduate education options may create barriers for members of rural and under-resourced communities, as well as those already in the workforce.

As workforce planning locally, regionally, and nationally now shifts toward a post-COVID-19 period, the pipeline from academia to practice is primed for redevelopment. Indeed, COVID-19 has taken its toll on the public health workforce, with two of three public health professionals in a recent national survey reporting experiencing burnout (Stone et al., 2021). This is on the heels of findings showing the national public health workforce to be aging and moving toward retirement. If all staff considering leaving or planning to retire did so, approximately 9,000 of the nearly 22,000 MCH staff would depart, taking an estimated 140,000 years of experience with them (Leider et al., 2016). It is not yet clear how COVID-19 has impacted turnover, although it is almost certainly to be higher than previous periods (Stone et al., 2021). Therefore, it is crucial that the field of MCH develop a national succession strategy that would assure that (1) state and local departments of health have capacity building and workforce development plans; and (2) that MCH practitioners have consistent, incremental leadership development opportunities throughout their career, from their entry into public health to the top levels of governmental and non-profit agencies serving MCH populations.

### Looking forward

The time is now for a multi-faceted recruitment and retention strategy to ensure an adequate and prepared MCH workforce with informed leadership and frontline staff to address the increasing needs of MCH populations both at state and local departments of health. This strategy would be reflective of findings from PH WINS and supported by HRSA via priorities such as in assuring a competent and diverse workforce that is able to address current and emerging needs (HRSA, 2019). This aligns with the 2021 MCHB strategic plan goal of strengthening public health capacity and workforce for MCH, and the specific objective of “strengthening state and local MCH agency capacity and infrastructure to provide and sustain the 10 essential public health services” (Ramos et al., 2022). Further, the strategy should aim to address critical barriers to a strong workforce, such as equitable pay, opportunities for advancement, continuing education and training, interpersonal and policy structures, inconsistencies in funding for governmental and non-profit agencies, and other facilitators of long-term retention and satisfaction (AMCHP, 2019). Natural funding streams for such an initiative could come via the Centers for Disease Control and Prevention and, perhaps most logically, the MCHB Division of MCH Workforce Development (DMCHWD). DMCHWD facilitates the training of MCH professionals to become leaders who can plan and administer programs that address the complex needs of MCH populations through goals of interdisciplinary training; advancing diversity and healthy equity; supporting workforce and leadership development; and advancing science, innovation, and quality improvement (Q.I.). Such a strategy should also aim to accomplish the following functions:

- Examine the MCH workforce in state and local departments of health using other sources of data and definitions to provide a more inclusive characterization of the MCH workforce.
- Enhance the capacity and skills of the MCH workforce in local departments of health given the training gaps of this sector of the MCH workforce.
- Create and strengthen pathways to link MPH students trained in MCH with local and state departments of health to increase the number of public health professionals specifically trained to serve MCH/Title V populations. Two priority areas include reinforcing local health departments serving rural areas given the unmet needs of rural communities (Kozhimannil et al., 2017; Probst et al., 2018; Richman et al., 2019) and connecting LHDs to other agencies to fill service gaps in certain MCH-related services.
- Focus on trainees of MCHB-funded Centers of Excellence in MCH who may become future leaders in the MCH field.

## Conclusions

Findings from this analytic essay suggest that academic institutions and governmental public health should collaborate in a shared recruitment and retention strategy, such as described above, to assure a qualified workforce and to strenghtn the MCH public health capacity and infrastructure (Ramos et al., 2022). Attention should be given to workforce development and capacity building in local departments of health where fewer trained MCH public health professionals are located. In order to address the underlying causes of health disparities that affect MCH populations, training is needed to prepare current and future MCH professionals to carry out critical public health tasks as outlined in the MCH Pyramid of Services, including needs assessment; program planning, administration, and evaluation; surveillance and monitoring; Q.I.; research; and policy, systems, and environmental change strategies (AMCHP, 2008). Critical to training efforts, such as those offered by the DMCHWD, is the need to incorporate training frameworks to identify and address the profound impact of racism on the health of mothers, children, and families (Trent et al., 2019; Gee & Ford, 2011; Jones et al., 2008). These priorities directly translate to the need for a highly skilled workforce able to develop, implement, and evaluate MCH programs and services.
